# Human resource and governance challenges in the delivery of primary eye care: a mixed methods feasibility study in Nigeria

**DOI:** 10.1186/s12913-021-07362-8

**Published:** 2021-12-10

**Authors:** Ada Aghaji, Helen E. D. Burchett, Ngozi Oguego, Shaffa Hameed, Clare Gilbert

**Affiliations:** 1 Department of Ophthalmology, College of Medicine, Enugu, Nigeria; 2 Department of Clinical Research, London School of Hygiene & Tropical Medicine, London, UK; 3 Department of Public Health, Environments and Society, London School of Hygiene & Tropical Medicine, London, UK; 4 Department of Ophthalmology, University of Nigeria Teaching Hospital, Enugu, Nigeria; 5 International Centre for Evidence in Disability, London School of Hygiene & Tropical Medicine, London, GB UK

**Keywords:** Primary eye care, Health workforce, Governance, Feasibility, Nigeria, Word count 5383.

## Abstract

**Background:**

To increase access to eye care, the World Health Organization’s Africa Region recently launched a primary eye care (PEC) package for sub-Saharan Africa. To determine the technical feasibility of implementing this package, the capacity of health systems at primary level needs to be assessed, to identify capacity gaps that would need to be addressed to deliver effective and sustainable PEC. This study reports on the human resource and governance challenges for delivering PEC in Anambra State, Nigeria.

**Methods:**

Design: This was a mixed methods feasibility study. A desk review of relevant Nigerian national health policy documents on both eye health and primary health care was conducted, and 48 primary health care facilities in Anambra state were surveyed. Data on human resource and governance in primary health facilities were collected using structured questionnaires and through observation with checklists. In-depth interviews were conducted with district supervisors and selected heads of facilities to explore the opportunities and challenges for the delivery of PEC in their facilities/districts. Data were analysed using the World Health Organization’s health system framework.

**Results:**

A clear policy for PEC is lacking. Supervision was conducted at least quarterly in 54% of facilities and 56% of facilities did not use the standard clinical management guidelines. There were critical shortages of health workers with 82% of facilities working with less than 20% of the number recommended. Many facilities used volunteers and/or ad hoc workers to mitigate staff shortages.

**Conclusion:**

Our study highlights the policy, governance and health workforce gaps that will need to be addressed to deliver PEC in Nigeria. Developing and implementing a specific policy for PEC is recommended. Implementation of existing national health policies may help address health workforce shortages at the primary health care level.

**Supplementary Information:**

The online version contains supplementary material available at 10.1186/s12913-021-07362-8.

## Introduction

Globally, it is estimated that 338 million people are blind or severely to moderately visually impaired [1]. Over 90% of the causes of vision loss are potentially avoidable e.g., cataract and refractive error, and over 90% of those affected live in low- and middle-income countries (LMICs) [2]. The prevalence of blindness increases with advancing age and is highest in those aged 50 years and above [3]. The estimated global prevalence of blindness is 0.49% among all ages, while the estimate for older adults (> 50 years) is at least 1.82%. Sub-Saharan Africa has the highest prevalence of blindness in older adults, which is estimated to be 4.19% in males and 4.36% in females [4]. In addition to conditions associated with vision loss, other less serious eye conditions like allergic and infective conjunctivitis are very common, and require appropriate management [5–7].

Much of the regional and gender variation in the prevalence of visual impairment and blindness is explained by inequity in access to eye care, particularly by the poor and women. Access to eye care services in sub-Saharan Africa is limited [8]. In addition, eye care in most LMICs is principally delivered at secondary and tertiary levels, in urban areas [9] leaving rural populations under-served. The World Health Organization’s (WHO) Global Action Plan 2014–2019 [10] and the World Report on Vision [9] advocate integrating eye care into primary health care (PHC) as a component of Universal Health Coverage, which could contribute to reducing this inequity in access.

The International Agency for the Prevention of Blindness (IAPB) defines PEC as “an integrated, participatory and inclusive approach to the eye health component of PHC consisting of promotive, preventive, curative and rehabilitative services.” [11] One of the challenges of delivering PEC in SSA has been lack of clarity on the scope of practice [12]. To address this, the WHO Africa Regional office recently developed and launched a package of PEC interventions for sub-Saharan Africa (WHO AFRO PEC) [13] to equip PHC workers, individuals and communities in sub-Saharan Africa to effectively manage common eye diseases [14]. This package, hereafter referred to as the WHO AFRO PEC package, [13, 14] has two broad components - eye health promotion and facility-based eye care. The eye health promotion component has two elements: 1) sets of health messages for children, mothers and care givers, and people of all ages and 2) information on how to give a health talk. For facility-based care there are six elements: 1) five evidence based algorithms (for red eye, eye swelling, trauma, vision loss for distance and near, and for children 0–5 years), 2) a set of 18 evidence based protocols covering several areas (measuring visual acuity (VA); applying an eye pad; prescribing medication; making referrals; removing foreign bodies, counselling, giving health talks), 3) a training package (curriculum and materials), 4) core lists of essential consumables, technologies and medicines, and 5) charts and recording forms. The purpose of the package is to strengthen the capacity of PHC workers in sub-Saharan Africa to manage patients with eye conditions [13] and widen access to eye care [15]. The package has been pilot tested in Rwanda and Kenya and has the potential to transform eye care in terms of treatment coverage for the majority of people with eye conditions in Africa [15].

However, PEC can only be as effective as the PHC elements of the health system into which it is integrated. In Nigeria, PHC is delivered in health centres and health posts, the former being larger and better equipped than the latter. Health centres provide 24-h services, including antenatal care and deliveries, while care health posts is more limited in scope.

The staff who work in PHC facilities and in the community include Community Health Officers (CHOs), Community Health Extension Workers (CHEWs), Junior Community Health Extension Workers (JCHEWs) and nurse-midwives (NMWs). Some primary health centres employ doctors (Additional File 1.) Support staff include health attendants and security staff [16]. The National Primary Health Care Development Agency (NPHCDA) is the national body which manages PHC and it has developed norms for staffing levels, supervision activities and minimum standards for the delivery of PHC [16]. Staff in PHC facilities are supervised by the local government authority (i.e., districts supervisors for health).

The purpose of this study was to assess the feasibility of integrating the WHO AFRO PEC package into the health system at PHC level in Nigeria, to allow policy makers and planners to make informed decisions about how the health system needs to be strengthened to deliver PEC [17]. Governance, financing and human resources for health (HRH) have been identified as key components for the successful implementation of health interventions [18]. This paper reports the findings of a mixed methods study of PHC facilities in Nigeria, highlighting the workforce and governance capacities to deliver PEC. Findings on equipment, service delivery and management information systems are reported in a companion paper.

## Methods

Snowdon describes many components of feasibility which include cultural, financial, technical and legal feasibility [19]. In this paper, we report some of the findings of a study to assess the technical feasibility of integrating eye care into PHC. The full protocol has been described elsewhere [20]. Based on Gericke’s technical feasibility framework, [21] the initial step was to develop a technical feasibility framework and then a technical capacity framework for PEC in sub-Saharan Africa, [22] from which tools were developed to assess the capacity of PHCs in Anambra state to deliver the intervention. Methods included a desk review, a facility survey, and semi structured interviews with relevant health workers.

### Desk review

A desk review of relevant Nigerian national PHC policy and national health policy documents was undertaken to assess the extent to which policies are in place to support the technical capacities required to implement the WHO AFRO PEC package (Additional File 2). These documents were obtained from the Federal Ministry of Health, the Primary Health Care Systems Development Department of the NPHCDA and the National Eye Health Strategic Team. Data of relevance to governance and the health workforce for the facility-based management component of the PEC package were mapped onto the WHO’s health systems framework [23].

### Facility survey

#### Study area

**Fig. 1:**
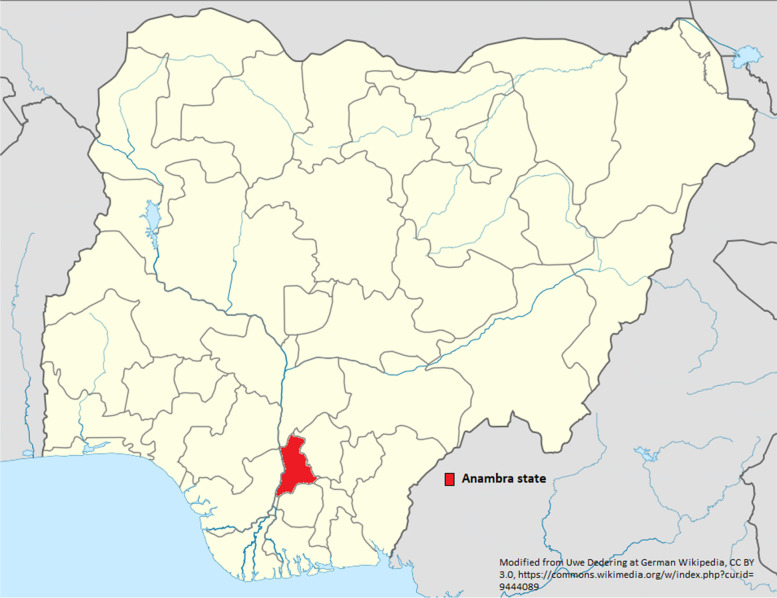
Map of Nigeria showing Anambra state

The study area chosen was Anambra state in southeast Nigeria, (Fig. 1) which has a population of 5.53 million [24]. There are 21 districts, which are classified by the government as rural, semi urban and urban, although there is some overlap. There are two tertiary hospitals, 35 secondary hospitals, and 347 PHC facilities comprising 235 health centres and 112 health posts (a ratio of 2:1). Anambra state was selected because it is one of the states in Nigeria that is yet to implement PEC [25].

#### Sampling PHC facilities

PHC facilities were selected using a two-stage stratified random sampling method to ensure representation by location and type of facility. In step one, six districts were selected randomly from a sampling frame of districts stratified by location so that two rural, three semi-urban and one urban district were selected. In step two, a list of all the PHC facilities in each selected district, stratified by type, was obtained from the National Primary Health Care Development Agency (NPHCDA). Forty-eight health facilities were proportionately selected i.e., more PHC facilities were randomly selected in districts with a larger number of facilities, maintaining the 2:1 ratio of health centres to health posts. Hence 33 health centres and 15 health posts were selected.

Data collection tools were designed based on two frameworks (one for health promotion, the other for facility-based care) developed using a Delphi exercise to assess the technical complexity of the different elements of PEC and the capacities needed for implementation [26]. Study tools included: 1. two observational checklists to assess the resources available, one for heads of facilities (CHOs, NMWs, or (J)CHEWs) and one for (J)CHEWs; 2. two structured questionnaires; one for each head of facility and one for a (J)CHEW/facility, and 3. topic guides for semi-structured interviews with selected heads of facilities and supervisors in each district. Based on a preliminary analysis of facility survey data, nine facilities were purposively selected (six health centres, three health posts) to represent high and low performing facilities in terms of workforce, supervision, available infrastructure, and patient visits /1000 population/year. In these facilities, facility heads were interviewed. The purpose of the interviews was to explore participants’ perspectives on the opportunities and challenges for delivering PEC in their facilities/districts.

The study team comprised the principal investigator (AA) and two research assistants from the Department of Ophthalmology, University of Nigeria: a recently qualified fellow in ophthalmology and an administrative officer. The principal investigator trained the team for two days on the study protocol, facility survey methodology, participant recruitment, data collection and ethical procedures. The principal investigator administered the checklists and structured questionnaires to facility heads and interviewed the supervisors and facility heads.

#### Data management

Quantitative data were entered by the principal investigator into databases created in Microsoft Access® for each questionnaire and checklist. Data were transferred to STATA V.15.1 (Statcorp, Texas) for analysis using STATransfer. Data were benchmarked against published norms, when available, such as the minimum number of each cadre of staff in health centres and health posts [16]. Simple descriptive analyses were performed e.g., the proportion of facilities visited with tools for referrals.

All interviews were conducted in English in the interviewee’s office in the presence of a trained note-taker. The interviews were recorded with an MP3 player after permission was granted. Interview methods and measures to ensure confidentiality are described in detail in a protocol paper [20].

The recordings were transcribed verbatim by AA and checked for accuracy by simultaneously reading the transcripts and playing back the audio recordings. The transcripts were read several times for familiarization with the content, and then coded using the WHO’s health system building blocks as the framework for analysis [26]. (Additional File 3). Framework analysis was used to obtain a descriptive overview of the data [27]. Initial coding of the transcripts was undertaken by AA using (Open Code Software V. 4.02) which was discussed with CG and HB. A matrix was developed to chart the data using the WHO Health systems building blocks in columns and the individual participant’s responses in rows [27]. To interpret the data, associations and comparisons were made within and between participants to generate themes.

##### Ethics

Ethical approval was obtained from the ethics review boards of the Federal Ministry of Health, Nigeria, the University of Nigeria Teaching Hospital, and the London School of Hygiene & Tropical Medicine. Permission was obtained to collect data from the State Ministry of Health and district departments of health. All participants gave written informed consent including to take photographs, [28] record interviews and use anonymous quotes where appropriate. Each participant was given a unique code to maintain confidentiality.

## Results

### Study sites

The health centres and health posts were selected from rural, semi- urban and rural districts in Anambra state (Table 1).

**Table 1:** Number of primary health facilities per districts selected for the study by probability proportionate to size

District location	Total health centres	Number selected	Total health posts	Number selected	Total selected
Rural	6	4	11	5	9
Rural	10	7	4	1	8
Semi-urban	11	6	2	1	7
Semi-urban	4	2	5	2	4
Semi-urban	5	3	7	3	6
Urban	20	11	5	3	14
**Total**	**56**	**33**	**34**	**15**	**48**

### Facility survey and interviews

Thirty three of the 48 PHC facilities visited were health centres and 15 were health posts. All facility heads were female, and their ages ranged from 37 to 63 years (mean 48.8 years). The average number of years spent as head of facility was 7 (range 1–22) years and 69% lived in the local community. Interviews were held with a range of staff, from community health extension workers to medical doctors (Table 2).

**Table 2:** Code numbers and characteristics of health workers interviewed

Identification code	Age group (years)	Sex	Qualification	Facility type	Location
**Heads of facility (HoF)**					
HoF/HP/U/1	51–60	Female	Com. Health Extension Worker	Health Post	Urban
HoF/PHC/U/2	31–40	Female	Nurse midwife	Health Centre	Urban
HoF/PHC/U/3	31–40	Female	Community Health Officer	Health Centre	Urban
HoF/PHC/SU/4	51–60	Female	Nurse midwife	Health Centre	Semi urban
HoF/PHC/R/8	31–40	Female	Community Health Officers	Health Centre	Rural
HoF/HP/R/7	31–40	Female	Com. Health Extension Worker	Health Post	Rural
HoF/HP/SU/6	41–50	Female	Community Health Officers	Health Post	Semi urban
HoF/PHC/SU/5	41–50	Female	Nurse midwife	Health Centre	Semi urban
HoF/PHC/R/9	51–60	Female	Nurse midwife	Health Centre	Rural
**Supervisors for health (SUP)**					
SUP/1	51–60	Male	Medical Doctor	*see below	
SUP/2	51–60	Female	Community Health Officers		
SUP/3	51–60	Male	Medical Doctor		
SUP/4	51–60	Male	Medical Doctor		
SUP/5	51–60	Female	Community Health Officers		
SUP/6	51–60	Male	Medical Doctor		

*The location of supervisors for health have been anonymised to protect their identity

### Human resources for health

#### Number of staff in the facilities

##### Health centres

None of the health centres met the approved norms for clinical staffing. The mean number of nurse/midwives per health centre was 1.1 (range 1–3), whereas the normative standard is 4 (Table 3). Over three quarters (27/33, 82%) of health centres had less than 20% of the total number of community health workers recommended, five had 30–60% of the total number of staff recommended and one had 90%.

**Table 3:** Staffing norms and number and cadre of clinical staff employed in health centres and health posts

	Health centres (***n*** = 33)			Health posts (***n*** = 15)			Total (***N*** = 48)
	*Norm	Mean (range) of available staff	% meeting norms	*Norm	Mean (range) of available staff	% meeting norms	% meeting norms
Doctors	1	0.24 (0–1)	24%	0	**NA	*NA	24%
Nurse midwives	4	1.1 (0–3)	0%	0	0.07 (0–1)	*NA	*NA
(J)CHEW / CHO	10	1.9 (0–9)	0%	1	1.6 (0–4)	93.7%	29%

*Norm = This represents the normative standards for both health centres and health posts in Nigeria [16]

**NA = not applicable as health posts are not required to be staffed by doctors or nurse midwives

##### Health posts

Some health posts were inappropriately staffed with nurse midwives or had up to four times the required number of community health workers (Table 3). 47% of health posts had the full complement of the clinical staff required, and 53% had more staff than recommended.

Shortage of staff in health centres was acknowledged by two supervisors, who said the following:

*You see, …. in all of the 34 health facilities [in the district] we have six nurses….. Six nurses!* SUP/3.

*None here has the full complement. In short, some of my so-called primary health centres have only one member of staff.* SUP/6.

The maldistribution of human resources affected the workload, as staff in health posts appeared to be under-utilised, as expressed by the head of a health post:

*Well, I would say that the work here is, let me tell the truth…it [the work] is not much, you understand.* HoF/HP/U/1.

However, health centre staff felt overworked, as a comment by a head of a health centre shows:

*We are already overloaded with work.* HoF/PHC/U/2.

The staff shortages leading to staff being overworked was also noted by the supervisors:

*Sometimes, things don’t go the way we like, but we cannot blame the health workers because they are handicapped in terms of manpower….to cope with the workload, most of them [heads of facility] employ volunteers. SUP/2.*

*It [the workload] is heavy ….the staff work long hours…..we hope that the government will one day recruit more staff. SUP/3.*

*You have some facilities that have too much work and some that are not doing anything. It depends on many things. Some facilities are sited where the community cannot reach it. SUP/5.*

An unexpected finding was that there was an almost equal number of volunteers and ad hoc staff working in the study facilities as formally employed PHC staff. Volunteers are either trained community health workers or informally trained health attendants who are not officially employed. Ad hoc workers, who are graduates in a medically related topic, are employed by the federal government on a temporary basis (1–2 years) and are paid the national minimum wage. Volunteers and ad hoc workers comprised 48.4% of those working in the facilities (Table 4).

**Table 4:** Health workforce in primary health centres facilities

Staff status	Staff in Health Centres		Staff in Health Posts		Total in PHC facilities	
	N	%	n	%	N	%
Employed	87	51.2	25	53.2	112	51.6
Volunteers						
(J)CHEWs	14	8.2	3	6.4	17	7.8
Health attendants	25	14.7	8	17.0	33	15.2
Ad hoc workers	44	25.9	11	23.4	55	25.4
Total	170	100.0	47	100.0	217	100.0

Heads of facility generally used volunteers to compensate for the lack of formally employed staff:

*Hmm….my workload is very hectic. But I have voluntary* [a volunteer]. HoF/PHC/U/3.

*You have seen it, I am the only person here, so I looked for someone to help me…. I have a volunteer.* HoF/PHC/SU/4.

Concerning the scope of work performed by the ad hoc workers, a head of facility said:

*It depends on their field and their training. We have a nurse who helps us to work and a microbiologist who does some statistics for us.* HoF/PHC/U/2.

The use of volunteers to overcome the critical shortage of staff appears to have been approved in principle by the local administrative authorities, but facility heads need to find money to pay them, as explained by a head of facility:

*Because the WDC [ward development chairperson] was told that, because there is a lack of manpower, each facility should employ one or two volunteers, but not more than two, from which we will source out funds to pay them. So, from there we manage to give them some stipend.* HoF/PHC/SU/5.

##### Staff turnover in the previous year

Two facilities had each employed a doctor in the previous year, and one had left. Five NMWs had been employed but ten had left, and 12 CHEWs had been employed while 16 had left. Hence in the previous year, there had been a net gain of one doctor and a net loss of five NMWs and four CHEWs from the facilities in the study. It appears when new facilities open, existing staff from other facilities are redeployed. This may account for staff turnover, as recounted by a facility head:

*You know how people are employed - today they open a health post, tomorrow again they open another facility. So, if you are three, they take one and post out [to another facility], take another and post out. That is the problem I have.* HoF/PHC/SU/4.

It also appears that there is also regular turnover of ad hoc staff, as suggested by another facility head:

*There is a saying that goes “soldier go, soldier come”. So, this one now…. she will finish by June (next month). When another batch [of adhoc staff] comes, there may be a nurse. If we request, the HOD [supervisor] will give us one.* HoF/PHC/U2.

##### Health worker training

In over 95% of facilities, there was at least one health worker who had received in-service training within the previous two years. The focus of the training was data collection and data management (78.8% of health centres and 66.7% of health posts), child health (76% of health centres and 87% of health posts), and maternal health (61% of health centres and 60% of health posts). Almost one in five (18.8%) facilities had workers who had undergone training in other areas of health, such as HIV. None had received in-service training in eye health, but 13.6% of (J)CHEWs reported having undergone pre-service training in eye care. The majority of the (J)CHEWs (93%) were willing to be trained in eye care. Staff had received no in-service training in care of the elderly and diabetes care. 60.9% of all the in-service training was supported by non-governmental organizations.

Comments by the head of a health centre suggested that staff attend several training sessions.

*Everyone gets trained. We go for a lot of trainings. Even recently there were some people that went for training on HIV. Some people have gone for training in reproductive health. So, we go for many trainings.* HoF/PHC/R8.

Concerning training in eye health, the head of a health post responded:

*Eye? We have not gone for training in eye.* HoF/HP/R7.

A comment by a head of facility suggested that volunteers also attend government funded in-service training.

*You know…. training always comes in batches….so they may call for one training, we may send our volunteer to go. So that is how we are doing it, it’s not always the officer in charge [that goes]….* HoF/PHC/SU/5.

Informal, on the job training of volunteers by facility heads was also reported, so that these staff could become useful. One head of facility said:

*So, I’ve been training my volunteer……and she’s picking up very fast. So, it’s helpful to me, she’s learning how to do it little by little*. HoF/PHC/R8.

##### Supervision

Regular supervision was conducted at least quarterly in over half of the facilities, and this was more common in health centres. However, visits by supervisors were not regular in a third of health centres and almost half of health posts. The majority (85%) of staff reported that data monitoring was the most common activity performed by supervisors during their visits (Table 5). Other supervisory activities such as teaching, observation of case management and feedback were performed less often.

**Table 5:** Regulation of primary health care activities

		Health Centre		Health post		Total	
		**N**	**%**	**N**	**%**	**N**	**%**
**Frequency of supervision in the previous 12 months**							
Norms met	Monthly	13	41.9	5	29.4	18	54.2
	Quarterly	6	19.4	2	11.8	8	
Norms not met	Bi-annually	1	3.2	2	11.8	3	6.2
	Irregularly	11	35.5	8	47.0	19	39.6
**Supervisors Activities**							
Data monitoring		28	84.8	13	86.7	41	85.4
Check supply of medications		14	42.4	8	53.3	22	45.8
Check supplies of other consumables		7	21.2	3	20	10	20.3
Teaching		11	33.3	5	33.3	16	33.3
Observe case management		14	42.4	5	33.3	19	39.9
Gives feedback		16	48.5	8	53.5	24	50
*Standard Operating Procedures (SOPs)							
Available and observed		12	36.4	7	46.7	19	39.6
Reported use of SOPs							
Norms met	Always	0	0	2	11.8	2	43.8
	Often	3	9.7	3	17.6	6	
	Sometimes	10	32.3	3	17.6	13	
Norms not met	Rarely	16	51.6	8	47.1	24	50.0
	Never	2	6.4	1	5.9	3	6.2

* SOPs are called National Standing Orders and are clinical guidelines for primary health care workers to manage patients with basic health conditions [29].

The supervisors were aware of the shortcomings of their supervision when so much training was required, as one commented:

*So (quarterly integrated) supportive supervision … essentially is training people on the job. Supervision wise, I say that we are not there. SUP/4.*

Another mentioned that there was an inadequate number of supervisors, and important challenges were lack of transport and the poor road infrastructure.

*The most pressing challenge is that of transport….transport. And then the personnel…. they are not enough ….it is also a problem. For instance, you’ll be the only person supervising over 30 health facilities. It’s not easy, so that’s the big challenge……. and the terrain. You have areas that have very bad terrain*…. *That place when it rains, it’s very slippery…..* SUP/3.

Two supervisors acknowledged the major role that non-governmental organisations (NGOs) play in supervising specific activities:

*For immunisation campaigns, if UNICEF sponsors, they will come and supervise it….they go to the field to see what’s happening…* SUP/3.

*What makes it [the immunisation programme] successful is that they (supervisors) do supervision from time to time. Constant monitoring. They are always in the field, monitoring. If there is any gap in the supervision, the staff backslide. Constant monitoring of staff - looking into what they do, then it will run well.* HoF/HP/SU/6.

Commenting on the possibility of supervising PEC when it is implemented, two supervisors noted the importance of training:

*The person supervising eyecare will need some training so that he or she will know what to do.* SUP/1.

*[For PEC] you need to train some people as supervisors…. So the supervisor will be supervising the health care people to be sure they are doing the right things.* SUP/4.

### Governance

#### Facility oversight

In the past, PHC facilities were administered at local government area (LGA) level - the third tier of governance. In 2018 administration was handed over to a state level agency, the Anambra State Primary Health Care Development Agency (ASPHCDA), the second tier of governance. Some facility heads felt that this had brought some positive changes:

*Yes, there is a lot of difference…a lot of difference. Because before they [the supervisors] never entered this community. If someone was sent on supervision the person will stay at his/her post and do what they like….But now they are trying, they come here and see what the community is like.* HoF/PHC/R/8.

*These people are more involved in our work than before and the supervisors come more frequently, steady, steady, steady. So that is the difference, and it has made everyone to buckle up.* HoF/HP/R/7.

*The only thing I’ve seen since we changed to the agency, is work. We have more work to do, more tasks……..We don’t rest since we moved to the Agency, all the time we are working…..Supervision is every week. So, what is happening is that if in the past you were coming to work twice a week, now you have to come every day.* HoF/HP/SU/6.

However, supervisors have borne the brunt of the transitional period of change in governance, including a change in the financial management, as two of them explained:

*Then this thing we are having….because of this movement from the LGA service commission to the ASPHCDA. So, you go there - you don’t know who is in charge. That is the problem, even the staff, the OICs [officers in charge] are having problems. Where do they pay the funds generated? Where are they going to pay this money they are supposed to be paying?* SUP/1.

*It’s a hard thing. In fact, we are feeling it. Because the local government - if you run to them for anything, they will say, “You people are not with us anymore. You run to the Agency, they will say “We don’t have money for anything now”. There is nothing for us now. In fact, we are just looking.* SUP/5.

##### Oversight for eye care

Most supervisors did not know whether eye care had been included as part of PHC, as one supervisor for health commented:

*Hmm…. It’s not one of the components of primary health care, unless it has been recently added*. SUP /2.

##### Clinical management guidelines

Less than 40% of facilities had clinical management guidelines, also called standard operating procedures (SOPs) or standing orders, and their reported use was low (Table 5). The regular use of SOPs was significantly more common in facilities headed by CHO/(J)CHEWs (62.5%) than by NMWs (25%) It appears NMWs are not required to use SOPs to manage patients, as mentioned by a head of facility and a supervisor:

*I am a trained nurse; I am not supposed to use standing orders. I don’t have any need for it. I am a trained nurse who was well taught. It is the CHEWs that use the standing orders. How can a trained nurse use standing orders?* HoF/PHC/SU/4.

*Remember that it is CHEWs that use standing orders, not nurses.* SUP/6.

However, a head of facility who is a community health worker attested to the importance she placed on the SOPs when she said:

*The standing order is our Bible. It’s our legal leg. So, if you do anything outside the standing order and there is problem, you’ll go in for it. But if you do it according to the standing order, it is your legal backing…… I encourage my staff to always use it.* HoF/PHC/U/3.

##### Policy findings (Additional File 2)

The National Health Policy 2016 emphasises that PHC is the focus of national health development. Recently, NPHCDA developed a PHC policy which devolves the administration of PHC to the states, who have administrative and financial autonomy [30].

In 2016, the NPHCDA developed a minimum health care package for PHC and included eye health under the non-communicable diseases (NCD) umbrella, [31] while eye health was only included in any Nigeria National Strategic Health Development Plans in 2018. However, one of the objectives of the recent Plan II (2018–2022) is to eliminate avoidable blindness and reduce the prevalence of visually impairing conditions [32]. Key strategies and activities to implement this include integrating eye care services into existing national health programmes, and building capacity for eye care delivery at all levels, including the primary level. Similarly, the National Health Policy 2016 recently included eye care in its priority public health interventions, with an initiative to integrate eye care services into the existing national health programs [33]. To improve coordination of eye care services in the country, the establishment of a functional unit for eye health at the Federal and State Ministries of Health is planned [32]. However, eye health is not included in the policy document, National Guidelines for the Development of Primary Health Care System in Nigeria, developed by the NPHCDA, (the central decision making body for PHC), which lists ten components of PHC such as maternal and child, oral and mental health [34].

##### Policy for human resources for health

The Federal Ministry of Health and its parastatals have developed several policies on human resources for health of relevance to PHC. (Additional File 2). For example, one of the goals of the National Health Policy (2016) is to provide appropriate and adequate human resources for healthcare at all levels of the health system, [33] including PHC. Another government policy stipulates that there should be a minimum number, mix and skill sets in each facility type, and that cadres of workers should be matched to services based on their competencies [16]. A further policy document indicates that the PHC management team should develop a sustainable system for human resources for health advancement and capacity building [31].

The National Health Act (2014) provides a sustainable funding policy for human resources at PHC level. It mandates the development and implementation of a Basic Health Care Provision Fund with 10% of the fund dedicated to the development of human resources for PHC [35]. The in-service training of PHC workers is the responsibility of State Ministries of Health [31].

For PEC, the National Eye Health Strategic Plan (2014–2019) recommends in-service training in eye care for PHC staff through workshops and seminars on the identification and management of some basic eye care conditions [36].

##### Policy for governance

Standing orders (PHC management guidelines for clinical care) are compulsory for (J)CHEW/CHOs and it is advisable that nurses/midwives, doctors, dentists and dental assistants working in PHC use them [34].

## Discussion

The main objective of this study was to assess the technical capacities of PHC facilities to deliver the WHO AFRO PEC package, in order to identify capacity gaps that would need to be addressed. This paper reports the findings in relation to human resources and governance and is the first study to assess the capacity of PHC facilities against a PEC benchmark and highlights the gaps that would need to be addressed to effectively deliver PEC.

The main findings were a critical shortages of trained health workers, which has in part been met by volunteers and ad hoc staff, inadequate supervision in terms of the frequency and activities, the low use of SOPs in the majority of facilities, and a lack of a clear policy for PEC.

In our study, there was a maldistribution of staff which affected the workload. More than half of the health posts were overstaffed, while none of the health centres met NPHCDA normative standards for staffing. To compensate for workforce shortages, PHC facility heads engage paid volunteers. The volunteers include trained (J)CHEWs who had not been formally employed and informally trained health attendants. There is no government policy to support this, hence they are not on the government’s payroll, but are paid a stipend from facility earnings. A considerable number of ad hoc workers were also working in facilities. They are university graduates, with or without relevant healthcare qualifications, who are paid to do community service for two years to reduce graduate unemployment and address deficiencies in public services (the N-power scheme) [37]. Our findings on the use of volunteers are similar to those in PHCs in Akwa Ibom state in South-south zone of Nigeria where 17.4% of the clinical staff were trained CHEWs who were informally employed as volunteers [38]. A challenge of relying on volunteers with basic training is that it is associated with dysfunctional health systems [39]. However, the use of volunteers may be a cause or a consequence of dysfunctionality. The untrained PHC worker situation is similar to that in Malawi where a significant proportion of the PHC workforce were unofficial health attendants [40]. This will pose a challenge for the delivery of PEC as the WHO AFRO PEC curriculum is specifically intended for trained medical personnel. Hence, delivering PEC in inappropriately staffed health facilities is likely to have grave implications for the quality of the services provided.

Low staffing levels are likely in part to be due to attrition of staff, as more had recently left facilities than had been recruited. An explanation given for this was that the Federal Government of Nigeria is revitalising PHC, and rather than employing new staff, existing staff are being redeployed to newly commissioned facilities, further worsening the health workforce situation. The absence of a national policy on staff transfers and postings in the PHC system in Nigeria [41] may have led to the unregulated transfer of PHC staff. A shortage of staff has also been reported in other countries, and it has been estimated that PHCs in sub-Saharan Africa only have 10% of the recommended number of PHC workers [42]. The Nigerian government urgently needs to implement policies for sustainable solutions to address the health workforce shortage [39]. It is anticipated that the new governance structure of PHC systems will lead to state governments taking responsibility for the recruitment of an appropriate number of trained staff.

The in-service training of PHC staff in study facilities focused on maternal and child health, with no training on eye care, diabetes and the elderly. These are gaps that needs to be addressed as the prevalence of eye disease increases with age, and up to 10% of people with diabetes have sight threatening retinopathy [43]. In addition, the low number of trained staff means that in-service training in the WHO AFRO PEC package would not give good coverage and would put additional strain on existing staff. Another factor to consider is that in-service training usually depends on additional funding, often from non-government sources, as identified in this study, which can result in episodic training when funding is available. In addition, our study found that volunteers as well as formally employed staff were trained, which suggests that PEC services may not be sustainable.

In Nigeria, PHC facilities should be supervised at least quarterly [34] but this requirement was met by only half of the facilities in this study during the previous year. This finding needs to be seen in the context of a recent change in the agencies responsible for PHC, and our study suggests that supervision has improved since the change was made. Reviews have been mixed on the benefits of supervision in PHC facilities in terms of the quality care [44] [45]. A cluster RCT on enhanced supervision of PEC in three East African countries revealed that facilities with enhanced supervision were more likely to have functioning torches and visual acuity charts, and staff were able to measure visual acuity better than their routinely supervised counterparts. However, there was no difference between the two groups in the ability of staff to identify and manage common eye conditions [46]. This suggests that supervision of PEC may not significantly impact the management outcomes of PEC unless case management is also supervised. In this study there is anecdotal evidence that regular and more frequent supervision of PHC facilities is one of the benefits of the new governance structure (i.e., transition from district level to ASPHCDA). However, the transition has presented some challenges and supervisors appear to be caught in the middle. Implementers of policies should adopt appropriate strategies to ensure a more collaborative change management process to successfully implement any reforms [47]. In addition, we suggest that more research on the impact of supervision of PEC will be needed.

We found that supervision in Anambra state focused on data monitoring, and there is need to include problem solving, feedback and mentoring. Indeed, recent studies have suggested broad strategies to improve supervision outcomes which include changing supervisory practices to create a more supportive environment for primary care providers [48]. Our study suggests that supervisors will also need to be trained in PEC to provide effective supportive supervision, which is one of the technical capacity requirements for the delivery of PEC [22].

National Standing Orders / SOPs are clinical guidelines for primary health care workers to manage patients with basic health conditions [29]. PHC guidelines also advise that NMWs and doctors use SOPs to maintain uniformity of practices at PHC level [34]. However, our study suggests that NMWs may be unlikely to use SOPs for eye care as less than half of the facilities used SOPs, a finding which was more common in facilities headed by a NMW. A plausible explanation for this is that the use of SOPs are not part of their training, unlike the training of (J)CHEWs and CHOs [49]. This has implications for the delivery of the WHO AFRO PEC package, which is driven by clinical algorithms and depends on SOPs. Staff who are trained to use algorithms in clinical practice will be best suited to deliver the package i.e., (J)CHEWs and CHOs. However, the availability of SOPs is not synonymous with their use, as demonstrated in our study and in others in the region [50]. For example, the WHO’s Integrated Management of Childhood Illness (IMCI) is another algorithm driven intervention. However, health workers in PHC facilities in Tanzania rarely adhered to IMCI guidelines when managing critically ill children [51]. Nevertheless, our study shows that PHC facilities headed by (J)CHEWs and CHOs are more likely to have and use SOPs. Interventions to increase adherence to SOPs will be needed when the PEC package is introduced.

### Implications for policy

Clear policies exist for sustainable human resource development and adequate staffing for PHC in Nigeria, but these policies are not being fully implemented. If implemented, these policies will deepen the capacity of PHC facilities to deliver crucial health interventions, including PEC. However, there is no PHC policy on staff deployment to regulate the indiscriminate transfer of staff.

Overall, there appear to be enabling policies for PEC in Nigeria, but these are scattered across general policies for eye health and other PHC policies. There is need for a unified PEC policy, like mental health and oral health in Nigeria which have specific policies at PHC level [34]. The lack of a defined policy for PEC may affect the development and sustained implementation of PEC in Nigeria, as government support is essential [8, 25]. For example, a report from South Africa suggests that the absence of an integrated policy for eye health promotion may be responsible for limited promotion activities [52]. In contrast, the integration of mental health into PHC in Ethiopia had policy backing, with very high-level government support [53]. Securing government support and appropriate resources for eye health in LMICs will require strong stakeholder engagement at political and economic levels in ministries of health.

The national eye plan recommends in-service training in PEC for PHC workers.

However, a more sustainable solution which would give greater coverage and quality of PEC services would be to include the WHO AFRO PEC package in the pre-service training of all relevant PHC workers, possibly as a component of non-communicable diseases training or care of the elderly. This has been successfully implemented in Rwanda, leading to a regular supply of PEC trained nurses [54].

Nigeria needs a coherent government policy for PEC which will frame eye health in the context of PHC systems, while aligning with other PHC and eye health policies. Such a policy should address pre-service training, the use of clinical guidelines and adequate supervision. High level advocacy will be needed to implement existing PHC human resource policies and address retention using context specific strategies [55].

A strength of this study is that the technical feasibility assessment was based on tools derived from a technical feasibility framework validated by PEC experts in sub-Saharan Africa [22]. We expect that the tools will be applicable to other African countries with similar settings as Nigeria. This was a mixed methods study which provided broad insights into the problems encountered in the health sector, including gaps and inconsistencies in relevant policies [56]. The triangulation of data from multiple sources i.e., policy documents, observational checklists, in-depth interviews and structured questionnaires provided a deeper understanding, from multiple perspectives, of the challenges and opportunities of delivering PEC in PHCs. This study is timely as the World Report on Vision has recently recommended delivering eye care services at PHC as a component of integrated people-centred eye care, [9] which is endorsed by the Lancet Commission on Global Eye Health [2].

A limitation of the study is that it was not adequately powered to detect statistically significant differences between health centres and health posts. Another limitation of the study is that financial, legal and political feasibility, as described by Snowden [19], were not included in this study which focussed on technical feasibility [21].

## Conclusions

Our study highlights health workforce, governance and policy gaps that will need to be addressed to deliver PEC in Nigeria. Developing and implementing a specific policy for PEC is recommended and the implementation of existing human resource policies may help address health workforce shortages.

Addressing the gaps highlighted in our study may result in the more effective delivery of PEC, but further research will be needed to assess the impact of an appropriately trained and supported eye health workforce on PEC and ultimately whether PEC delivery can reduce the prevalence of blindness in sub-Saharan Africa.

## Supplementary Information

**Additional file 1.**

**Additional file 2.**

**Additional file 3.**

## Abbreviations

AFRO: African Region
ASPHCDA: Anambra State Primary Health Care Development Agency
CHEW: Community Health Extension Worker
CHO: Community Health Officer
IAPB: International Agency for the Prevention of Blindness
IMCI: Integrated Management of Childhood Illnesses
JCHEW: Junior Community Health Worker
LMICs: Lower Middle-Income Countries
NCD: Non-Communicable Diseases
NMW: Nurse Midwife
NPHCDA: National Primary Health Care Development Agency
PEC: Primary Eye Care
PHC: Primary Health Care
SOP: Standard Operating Procedure also called National Standing Orders
WHO: World Health Organization
