# Supplementary material for: Human resource and governance challenges in the delivery of primary eye care: a mixed methods feasibility study in Nigeria
Source: BMC Health Serv Res. 2021 Dec 10;21:1321. doi: 10.1186/s12913-021-07362-8 (PMC8662916; doi:10.1186/s12913-021-07362-8)
Supplement: Supplementary file 3 — Additional file 3. [file 12913_2021_7362_MOESM3_ESM.docx]

(1) World Health Organization. Everybody's business--strengthening health systems to improve health outcomes: WHO's framework for action. 2007

**Codes for data analysis of semi-structured interviews.**

|  | Code | Interpretation |
| --- | --- | --- |
|  |  |  |
|  | Health workforce | Any information relating to the Employment, Attrition, staff turnover, staff training, number and distribution of staff, staff workload, |
|  |  |  |
|  |  |  |
|  | Governance | Any information relating to Policy, Supervision, Discipline, Leadership, clinical guidelines, oversight and accountability |
|  |  |  |
|  | Information | Any information relating to data collection; staff and patient registers, data transmission and dissemination, referral data |
|  |  |  |
|  |  |  |
|  | Service Delivery | Any information pertaining to services delivered in the facilities or communities, types of interventions, target clients, location |
|  |  |  |
|  |  |  |
|  | Medical Products, Vaccines, Technology | Any information pertaining to the availability, procurement and maintenance of equipment and technologies and supply delivery mechanisms for consumables. |
|  |  |  |
|  |  |  |
|  | Financing | * Out of the scope of this research |
|  |  |  |

1. World Health Organization. Everybody's business--strengthening health systems to improve health outcomes: WHO's framework for action. 2007.
